# Prognosis of multi-unit implant supported and combined tooth-implant supported fixed dental prostheses: a retrospective cohort study with a mean observation period of 4.27 years

**DOI:** 10.1007/s00784-025-06548-2

**Published:** 2025-09-17

**Authors:** Moritz Waldecker, Peter Rammelsberg

**Affiliations:** Department of Prosthetic Dentistry, Heidelberg University Hospital, University of Heidelberg, Im Neuenheimer Feld 400, 69120 Heidelberg, Germany

**Keywords:** FDP, Long-span, Survival, Support, Units, Loading factor

## Abstract

**Objectives:**

The objective of this retrospective cohort study was to evaluate survival of multi-unit FDPs in comparison to 3-unit fixed dental prostheses (FDPs).

**Materials and methods:**

434 FDPs placed in 326 patients were selected from a prospective clinical long-term study. 213 FDPs were solely implant-supported, 154 FDPs tooth-implant supported, and 67 FDPs were cantilever FDPs. The most FDPs had 3-units (*n* = 315), 95 FDPs had 4-units, and 24 FDPs had more than 4 units. The most FDPs had a unit/abutment relation of ≤ 1.5 (*n* = 336), and 98 FDPs had a relation of > 1.5. Kaplan-Meier curves were used to estimate the survival probability of the FDPs for the variables type of FDP support, number of units and loading factor. Univariate log-rank tests were used to test for differences between groups within variables.

**Results:**

The mean observation period was 4.27 years. In the observation period of up to 12.6 years 17 FDPs failed mainly through technical complications. The underlying causes were implant loss (*n* = 6), abutment tooth loss (*n* = 5), loosening of the abutment screw (*n* = 1), and extensive chipping (*n* = 5). Survival probability of all FDPs was ≥ 89,6% after 10 years. Log-rank tests revealed no significant differences between groups for all variables (support, number of units, and loading factor (*p* ≥ .339).

**Conclusions:**

Because of their promising prognosis 4-unit FDPs placed on implants or a combination of tooth and implant can be recommended as an alternative to 3-unit FDPs. A higher number of implants in relation to FDP units does not improve the prognosis of FDPs.

**Clinical relevance:**

Support, number of units and the loading factor do not influence the survival of FDPs. Therefore, 4-unit FDPs placed on implants or a combination of tooth and implant are a valuable treatment alternative to 3-unit FDPs.

## Introduction

Due to improved prevention programs, patients today have less caries experience, more of their own teeth and are less frequently edentulous. As a result, the indication for minimally invasive treatment methods is becoming increasingly common. However, if several teeth need to be replaced and removable dentures are to be avoided, strategic implant placement is a reliable treatment option. Medium-term survival rates of 93 to 99% are reported for solely implant-supported fixed dental prostheses (FDPs) [1]. For anatomical reasons, it is not always possible to place several implants to support an FDP. Tooth-implant-supported FDPs represent a cost-effective and surgically minimally invasive treatment alternative. Promising 5- and 10-year survival rates of 77 to 96% and 72 to 78% have been reported for tooth-implant-supported FDPs [1, 2]. The combination of teeth and implants is still a controversial topic. Compared with solely implant-supported FDPs, tooth-implant-supported FDPs have a comparable survival rate. Rigid support either on implants alone or in combination with teeth is not decisive for the survival of FDPs [2, 3] and the chipping incidence [3]. On the other hand, non-rigid abutment tooth support can lead to unacceptable intrusion of abutment teeth [4, 5] and should therefore be avoided [6]. The chipping incidence of solely implant- and tooth-implant-supported FDPs depends significantly on the selected framework material and the veneer design. Compared to fully veneered noble metal, fully veneered zirconia has a 2.79-fold higher risk of chipping. At least medium-term data show that the risk of chipping can be significantly reduced by omitting the veneer in the load-bearing area [3].

Most studies report on solely implant- and tooth-implant supported FDPs with predominantly three to a maximum of four units [6]. However, if several gaps need to be closed and the abutment arrangement is unsuitable for several 3-unit FDPs, multi-unit FDPs are indicated. The literature lacks conclusive studies on the effect of the number of units and the relation of units to abutments in solely implant- and tooth-implant supported FDPs. In this context, however, it seems obvious that an unfavourable loading condition leads to a higher implant load in multi-unit FDPs and consequently has negative effects on implant survival.

Therefore, the objective of this observational cohort study was to investigate the survival of multi-span FDPs in comparison to 3-unit FDPs. The null hypotheses were that no difference would be found between the incidence of failure between FDPs with different support, that no difference would be found between the incidence of failure between FDPs with different number of units, and that the incidence of failure would not differ between FDPs with a different unit/abutment relation (loading factor).

## Materials and methods

This retrospective, single center cohort study was conducted in accordance with the World Medical Association Declaration of Helsinki and was approved by the local university study ethics commitee (registration number: 027/2005). All participants gave their informed and written consent to scientific use of the recorded data.

All implant-supported or tooth/implant-supported FDPs placed by members of the Department of Prosthodontics with an observation period of at least 6 months formed the primary data set. In the further selection process, 89 FDPs from a prospective clinical study of 523 documented FDPs could not be included for different reasons. Fifty-four FDPs with an observation period greater than 6 months were excluded because of missing follow-ups, for 12 FDPs the consent form was missing, and 15 FDPs were excluded because observation periods after prosthetic restoration were too short. In Table 1 an overview of reasons for exclusion is given in detail.

**Table 1:** Reasons for exclusion of eligible FDPs

Total number of eligible FDPs	523
Period after prosthetic restoration < 6 months	−15
Missing consent form	−12
Follow-up missed	−54
Refused to attend follow-up visit	−2
Prosthetic restoration elsewhere	−1
Moved away	−1
Died	−1
No restoration because of financial constraints	−1
Screw-retained FDPs	−2
Total	434

Inclusion criteria were signed informed consent forms, FDPs placed on implants or a combination of teeth and implants, a conventional loading protocol, and a minimum observation period of at least 6 months after placement of the FDPs. All implants were inserted by experienced dentists of the Department of Prosthodontics. A delayed loading protocol was used for the implants, ranging from 3 months after implant placement in the mandible to a maximum of 9 months for augmented sites in the maxilla. All FDPs were fabricated by 1 of 2 dental laboratories that had been calibrated in the fabrication procedures. Thus, a total of 434 FDPs placed in 326 participants (42,9% men) from 21 to 88 years of age (mean: 61.2 years) were examined.

Mainly tissue level implants (*n* = 363) or bone level implants (*n* = 43) from one manufacturer (Straumann AG) were used. The other implants were 28 Replace implants (Nobel Biocare Services AG). Predominantly implants with a diameter of 4.1 mm (*n* = 341) and a length of 10 mm (*n* = 342) with a range of 3.3 to 5.0 mm in diameter and 4 to 16 mm in length were used.

**Fig. 1:**
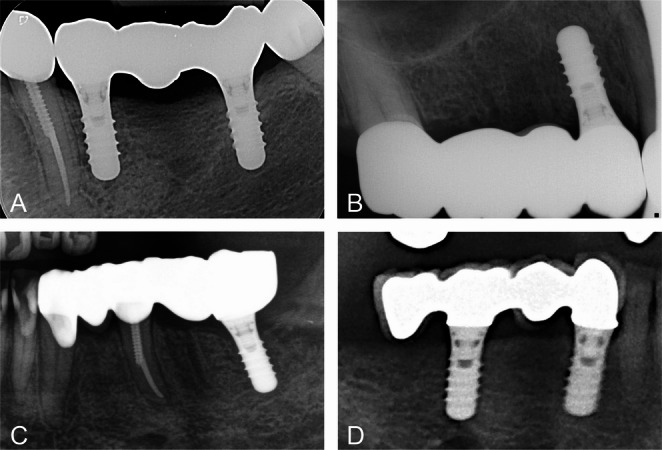
Radiographs of exemplary fixed dental prostheses (FDP). **A**, Implant supported FDP with 3 units and a unit/abutment relation of 1.5. **B**, Combined toot-implant supported FDP with 4 units and a unit/abutment relation of 2. **C**, Combined toot-implant supported FDP with 5 units and a unit/abutment relation of 1.67. **D**, Implant supported cantilever FDP with 4 units and a unit/abutment relation of 2

The FDPs were predominantly located in the posterior region (region from first premolar to third molar, *n* = 308), 46 FDPs were located in the anterior region (region from canine to canine), and 80 FDPs combined the anterior and posterior region. The FDPs included 213 implants supported FDPs, 154 tooth-implant supported FDPs, and 67 cantilever FDPs (only 1 tooth-implant supported FDP) with a maximal extension of 8 mm. The indication for use of natural abutment for tooth-implant supported FDPs did not differ compared with conventional FDPs. The most FDPs had 3 units (*n* = 315), 95 FDPs had 4 units, and 24 FDPs had more than 4 units. None of the FDPs with more than 4 units extended crossarch. The most FDPs had a unit/abutment relation of ≤ 1.5 (*n* = 336), and 98 FDPs had a relation of > 1.5. Figure 1 shows exemplary radiographs of investigated FDPs. All FDPs were cemented. 252 FDPs with a definitive cement and 119 FDPs with an interim cement. Further characteristics of FDPs are given in Table 2.

**Table 2:** Characteristics of FDPs

Variable	Characteristics	*N*
Support	Implant-implant	213
	Tooth-implant	154
	Implant cantilever	66
	Tooth-implant cantilever	1
Units	3	315
	4	95
	> 4	24
Loading factor	≤ 1.5	336
	> 1.5	98
Material	High nobel metal alloy, complete veneer	225
	Co-Cr base metal alloy, complete veneer	35
	Zirconia, complete veneer	43
	Zirconia, partial veneer	63
	Zirconia, monolithic	68
Attachment method	Definitive cement *Glas-ionomer cement* *Others*	252 *229* *23*
	Interim cement	119
Location	Anterior	46
	Posterior	308
	Combined Anterior - Posterior	80

As occlusion concept an anterior canine guidance was established if anatomically feasible. Care was taken to ensure that the pontics had no dynamic occlusal contacts.

Follow-ups were scheduled at 6-month intervals, and radiographs were made at 1-year intervals without the use of a positioning device. As part of the follow-ups, all teeth and restorations were inspected, oral hygiene training was carried out and teeth were professionally cleaned. The following were documented: probing depth, bleeding on probing, and occlusal contacts in static and dynamic occlusion. Any complications, for example loss of retention, fractures of abutment or frameworks, and chipping of the veneering ceramic, were also recorded on standardized forms.

Kaplan-Meier-Curves were calculated for FDP survival. Univariate log-rank tests were used to test for differences between groups within variables support, number of units, and loading factor. Statistical analysis was conducted with a statistical software program (SPSS) with α = 0.05.

The authors declare, that the manuscript is in compliance with the STROBE checklist.

## Results

The mean observation period was 4.27 years. In the observation period of up to 12.6 years 17 FDPs failed. Implant loss (*n* = 6), abutment tooth loss (*n* = 5), loosening of the abutment screw (*n* = 1) and extensive chipping (*n* = 5) were the underlying causes (Table 3 and 4).

**Table 3:** Reasons for failure of FDPs

Reason for failure	*N*
Implant failure	6
Abutment tooth loss	5
Caries	*2*
Endodontic complications	*2*
Fracture	*1*
Extended chipping	5
Abutment screw loosening	1
Total	17

**Table 4:** Characteristics of FDP failures

		Failure	
Variable	Characteristics	No	Yes
Units	3	303	12
	4	91	4
	> 4	23	1
Support	Implant-implant	205	8
	Tooth-implant	146	8
	Implant cantilever	65	1
	Tooth-implant cantilever	1	0
Loading factor	≤ 1.5	321	15
	> 1.5	96	2

**Fig. 2:**
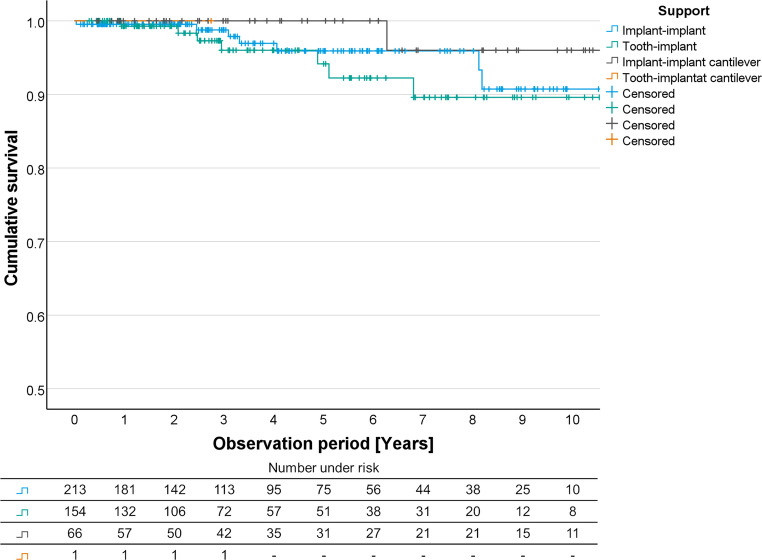
Kaplan-Maier survival curves for conventional implant-supported, combined tooth-implant-supported, implant-supported cantilever, and combined tooth-implant-supported cantilever FDPs. FDP, fixed dental prostheses

**Fig. 3:**
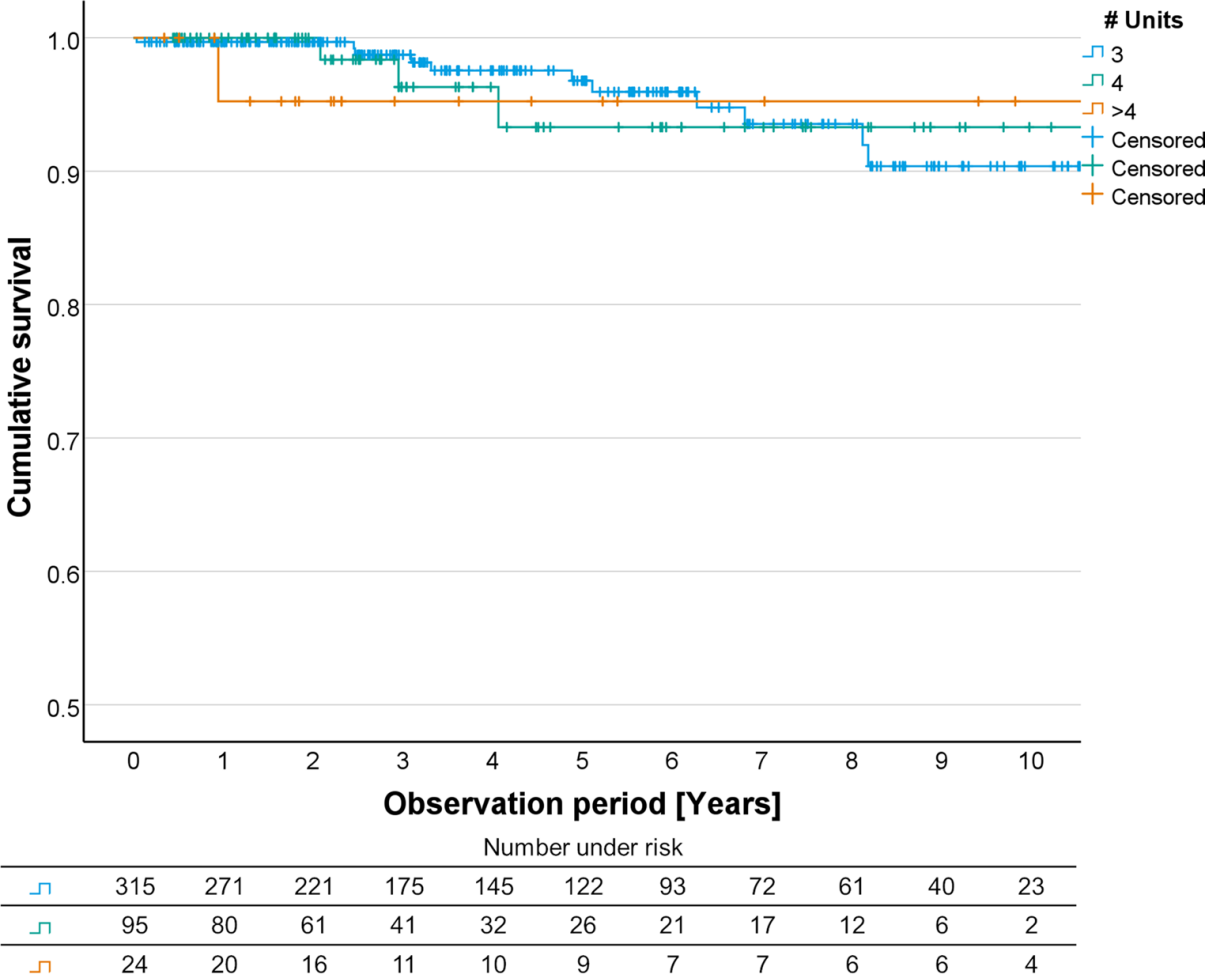
Kaplan Meier curves for FDPs with 3, 4, and more than 4 units. FDP, fixed dental prostheses

**Fig. 4:**
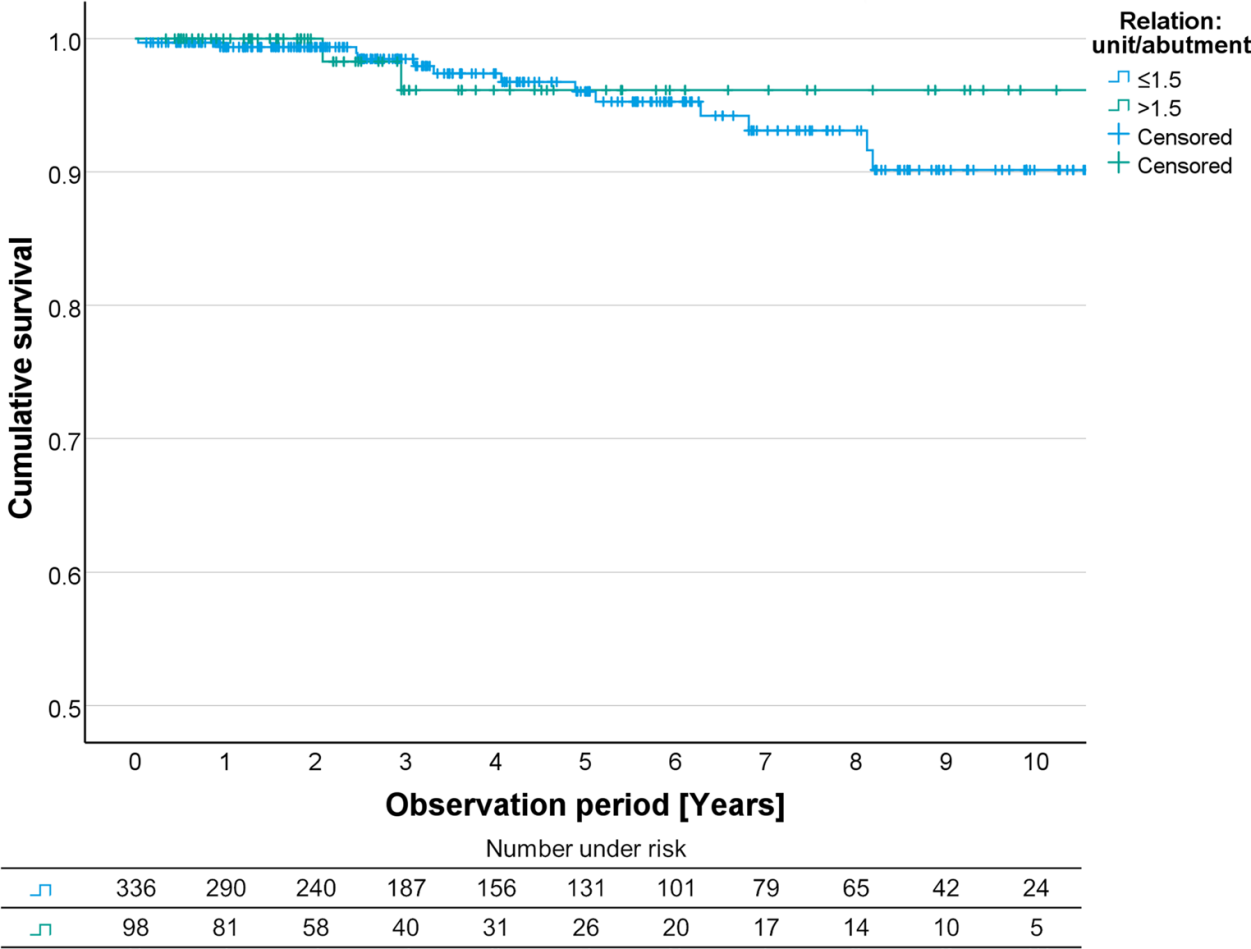
Kaplan Meier curves for FDPs with a unit/abutment relation of ≤1.5 and>1.5. FDP, fixed dental prostheses

All Kaplan-Meier curves for FDPs with different support, with a different number of units, and with a different unit/abutment relation showed overlaps between the individual levels (Figs. 2, 3 and 4).

The Kaplan-Meier survival analysis for FDPs with different support showed a survival probability of 95.9% after 5 years and 90.7% after 10 years for FDPs supported by solely implants, 94.2% and 89.6% for combined tooth-implant-supported FDPs, 100% and 96.0% for implant-implant cantilever FDPs (Fig. 2).

For FDPs with a different number of units the Kaplan-Meier survival analysis showed a survival probability of 96.8% after 5 years and 90.4% after 10 years for 3 units FDPs, 93.3% after 5/10 years for 4 units FDPS, and 95.2% after 5/10 years for more than 4 units FDPs (Fig. 3).

For FDPs with a different loading condition the Kaplan-Meier analysis revealed a survival probability of 96.0% after 5 years and 90.1% after 10 years for FDPs with a unit/abutment relation of ≤ 1.5, and 96.1% after 5/10 years for FDPs with a relation of > 1.5 (Fig. 4).

Log-rank tests revealed no significant differences between groups for all variables (support, number of units, and loading factor; *p* ≥.339).

## Discussion

The null hypotheses that no difference would be found between the incidence of failure between FDPs with different support or number of units, and that the incidence of failure would not differ between FDPs with a different loading factor was accepted.

17 FDPs were lost. This was mainly due to biological complications, including 6 implant losses and 5 tooth losses. Six additional technical failures were predominantly associated with extended chipping of ceramic veneers (*n* = 5). No significant risk factor for failure could be isolated. Neither the support, the number of units nor the FDP-unit/abutment relation had any influence on the survival probability of the FDPs.

The present study was unable to find an effect of support on the survival of the FDPs. A previous review reported a 10-year survival rate of 86.7% for solely implant-supported FDPs, whereas only 77.8% of combined-supported FDPs are still in situ [1]. In contrast, the present study found a 10-year survival rate of more than 89% for FDPs supported on teeth and implants. As mentioned before the combination of teeth and implants to support FDPs is seen controversially in the literature. Nevertheless, combined toot-implant supported FDPs are seen as a viable prosthetic treatment alternative to solely implant-supported FDPs [4]. Therefore, without a specific indication, teeth should not be extracted in favour of implants to avoid tooth-implant supported FDPs [7].

In the present study, the number of units had no effect on survival or the occurrence of complications. The majority of FDPs were 3-unit (*n* = 315), followed by 95 4-unit FDPs and 24 FDPs with more than 4 units. There are generally few studies in the literature that specifically investigate the influence of the number of units. Furthermore, there are only predominantly studies with long-span FDPs that are either solely supported by teeth or solely by implants. Studies on long-span FDPs supported by a combination of teeth and implants are rare. The results from these studies are partly in contrast to the results of the present study.

Alstertal et al. examined 78 solely tooth-supported FDPs with at least 5 units. After 10 years, the FDPs had a survival rate of 74.4% and a success rate of 52.6%. The most common complications were mainly technically: chipping (38%), loose retainers (7.7%), and framework fractures (3.8%). Biological complications were less frequent (caries (14%), endodontic complications (11.5%), and root fractures (5.1%)). Significantly more complications were observed in FDPs with post-and-cores or cantilevers, especially in combination. Most FDPs were metal-ceramic FDPs (Au (49), Co-Cr (16), Ti (6), ZrO2 (2), unknown (5)) [8].

Another study analyzed a total of 258 FDPs over a period of up to 15 years. Of these, 196 were short-span (3–4 units) and 62 were long-span (5 units and more) FDPs. A total of 74 purely technical complications occurred. Chipping (*n* = 66) was the most common complication followed by loss of retention (*n* = 11). In comparison with short-span FDPs, significantly more complications were observed for long-span FDPs over the long-term observation period of 15 years. The cumulative survival probability for short-span FDPs was 91% after 5 years, 68% after 10 years and 34% after 15 years. For long-span FDPs, the probability of survival was 85% after 5 years, 50% after 10 years and 18% after 15 years [9].

Nilson et al. examined 63 patients with a total of 86 FDPs over a mean observation period of 7.25 years. 33 FDPs were solely tooth-supported and 53 FDPs were solely implant-supported. 67 FDPs had 3–5 units and 19 FDPs had 6–12 units. FDPs with 6–12 units had a 5.27-fold higher risk of receiving an unacceptable rating. FDPs were considered unacceptable if they had a complication requiring intervention (polishing or remaking). No significant effect on survival and success was found for solely tooth-supported FDPs. In contrast, the risk of achieving an unacceptable rating was 23.3 times higher for solely implant-supported FDPs with 6–12 units. In addition, the risk of failure for these FDPs was 18.5 times higher than for FDPs with 3–5 units [10].

In the present study, an explicit distinction was made between FDPs with a unit/abutment relation of ≤ 1.5 or < 1.5 to investigate the effect of an unfavorable loading condition on the FDP survival. The authors are not aware of any studies that have investigated the influence of the unit/abutment relation for tooth-implant or purely implant-supported FDPs. Results from studies on cross-arch fixed prostheses according to the All-on-4 and All-on-6 concept show that an unfavourable loading condition does not necessarily have an influence on the survival of implant-supported restorations and corresponding implants. For cross-arch fixed prostheses according to the All-on-4 concept in the upper jaw, a prosthesis success rate of 99.2% is reported after 5–13 years [11]. A prosthesis survival rate of 98.8% is reported for prostheses in the mandible after 10–18 years [12]. Zhang et al. examined 217 patients with 1222 implants supporting 271 cross-arch fixed prostheses (202 prostheses supported by 4 implants, and 69 prostheses supported by 6 implants) over an observation period of 3–13 years. The results show an implant survival of 95.9% for the All-on-4 group, and 96.4% for the All-on-6 group. The prosthesis survival was 95.1% for All-on-4, and 94.2% for All-on-6. No significant difference was found between the two concepts [13]. The results found in this study show that a higher unit/abutment ratio has no influence on the survival of the FDPs examined. Thus, the decision in favor of a short- or long-span FDP is not dependent on the future span of the FDP, but is rather influenced by other factors such as physical principles, material and anatomical conditions. It can be concluded from the results that a higher number of implants does not improve the prognosis of an FDP. Minimally invasive treatment concepts for providing patients with FDPs supported by teeth and implants therefore appear to be a long-term treatment alternative.

There are some limitations to the study. The study itself was retrospective in nature. The FDPs examined were very heterogeneous in terms of material and veneer design. It has already been shown that the combination of material and veneer design has an influence on the occurrence of complications [3]. The number of cases with regard to support, number of units and abutment/unit ratio was unevenly distributed. Therefore, the lack of significant differences between groups may be due to low statistical power. Different clinical conditions such as the presence of bruxism and the quality of the antagonistic dentition can influence the performance of FDPs. However, these variables were not analyzed separately. Due to these limitations, the results of the present study should be interpreted with caution.

## Conclusion

Because of their promising prognosis 4-unit FDPs placed on two implants or a combination of tooth and implant can be recommended as an alternative to 3-unit FDPs. A higher number of implants in relation to FDP units does not improve the prognosis of FDPs.

## Data Availability

No datasets were generated or analysed during the current study.
